# Evaluation of amplicon-based nanopore sequencing for foot-and-mouth disease viruses in clinical and environmental samples

**DOI:** 10.1128/spectrum.00791-26

**Published:** 2026-07-28

**Authors:** Ji Hyun Jeon, Soyoon Ryoo, Hyeonjeong Kang, Jongwan Kim, Da-Rae Lim, Antonello Di Nardo, Andrew E. Shaw, Donald P. King, Su-Mi Kim

**Affiliations:** 1 Foot-and-Mouth Disease Diagnostic Division, Animal and Plant Quarantine Agency 65359, Gimcheon-si, Gyeongsangbuk-do, Republic of Korea; 2 The Pirbright Institute, Pirbright, Surrey GU24, United Kingdom; Changchun Veterinary Research Institute, Chinese Academy of Agricultural Sciences, Changchun, China

**Keywords:** foot-and-mouth disease virus, nanopore sequencing, whole-genome sequencing, large-scale evaluation, outbreak investigation

## Abstract

Foot-and-mouth disease (FMD) causes severe global economic loss, necessitating rapid viral characterization. Nanopore sequencing provides a simple, real-time workflow suitable for on-site outbreak response, addressing the limitations of conventional methods. In this study, we optimized a previously published amplicon-based protocol and used this method to characterize a diverse range of samples (vesicular fluid, epithelium, serum, nasal/oral swabs, and environmental samples) collected during FMD outbreaks in 2025 in the Republic of Korea. Of the 129 samples collected, we successfully recovered complete genomes from 37 samples and VP1 sequences from 85 samples. Amplifying the S-fragment in isolation and separately barcoding each pool of PCR amplicons markedly improved sequence recovery. Furthermore, sequencing success depended on viral load and sample type. Based on comparisons with real-time RT-PCR results, whole-genome sequence (WGS) recovery exceeded 77.3% at cycle threshold (Ct) values ≤25 across all clinical samples. In the Ct > 30 category, serum samples yielded the highest WGS recovery rates (44.4%). This rate was markedly higher than the success rates observed for epithelium (20.0%) and nasal swabs (9.1%), whereas oral swabs and environmental samples failed to yield any sequences (0%). However, VP1 recovery from environmental samples reached 80% at Ct ≤ 30 (8/10), providing an approach to enable non-invasive monitoring. These findings demonstrate that amplicon-based nanopore sequencing is a practical method for the rapid generation of genomic data during FMD outbreaks.

**IMPORTANCE:**

Although rapid detection and genomic data analysis are crucial for effective foot-and-mouth disease (FMD) control, the collection of these data can be challenging for certain sample types and impacted by reduced viral loads that result from nationwide FMD vaccination. This study provides a practical solution through large-scale evaluation of an optimized amplicon-based nanopore sequencing protocol to enhance the sequencing success rates for both clinical and environmental samples. Using a modified protocol to enhance genome recovery, we demonstrated that sequence data could be retrieved from diverse sample types (even with high real-time RT-PCR cycle threshold values). We identified serum as the most suitable sample, with environmental sample sequencing allowing for non-invasive monitoring during outbreaks. These results support the use of nanopore sequencing for rapid genomic analysis, particularly in outbreak responses, such as rapid surveillance, emergency vaccine selection, and epidemiological monitoring.

## INTRODUCTION

Foot-and-mouth disease (FMD) is a highly contagious viral disease affecting cloven-hoofed animals, causing severe economic losses worldwide due to trade restrictions and costly control measures such as culling and ring vaccination (1, 2). The rapid evolution of FMD viruses (FMDVs), which are classified into seven serotypes (O, A, Asia 1, C, Southern African Territories (SAT) 1, SAT 2, and SAT 3) as numerous constantly emerging lineages, necessitates precise and rapid genetic characterization for effective disease management (3, 4). Rapid sequence acquisition of the VP1 coding region is essential for initial genotyping of circulating viruses, tracking their geographic viral distribution, and enabling critical monitoring of potential risks for transboundary movements of novel and exotic threats (3, 5–7). Whole-genome sequencing (WGS) provides data that enable tracing of outbreak origins and understanding of transmission pathways at high resolution (6, 8, 9). Furthermore, genetic data for sequences encoding the FMDV capsid can be used to understand antigenic phenotypes and select appropriate vaccines for controlling outbreaks (10–12).

Whole-genome sequencing approaches typically rely either on first-generation sequencing technology, Sanger sequencing, or next-generation sequencing platforms (e.g., Illumina). Although Sanger sequencing offers high accuracy and medium read lengths, it is limited by its relatively low throughput and high costs when applied to large-scale genomic analysis (13, 14). Illumina platforms are widely used for their high accuracy; however, they are less suitable for obtaining rapid results, require accumulating numerous samples to be cost-effective, and have a fixed run time, which can delay access to critical data (15, 16). To overcome these limitations, long-read sequencing using Oxford Nanopore Technology (ONT) represents a paradigm shift for the genetic characterization of infectious diseases. The platform is portable and enables real-time data streaming, facilitating immediate analysis from the start of sequencing, which makes ONT uniquely suited for rapid emergency response and comprehensive genomic data generation (16–21).

Amplicon-based nanopore sequencing has been successfully used for the rapid genomic analysis of various pathogenic viruses, including Ebola virus (21), Zika virus (22), Lassa virus (23), and SARS-CoV-2 (24), highlighting its suitability in active disease outbreaks. Moreover, recent studies have demonstrated the potential of this technology for the analysis of FMDV (1, 25, 26). Notably, Brown et al. (1) described that nanopore sequencing could provide precise and rapid analysis of the P1 coding region from FMD clinical samples. Building on these results, Shaw et al. (26) designed primers to amplify the broad-spectrum amplification of the entire FMDV genome using two approaches: the S_ and L_schemes (for 20 short and 6 longer fragments, respectively). Although both methods effectively amplify the entire genome, the L_ scheme is more reliable and cost-effective, further displaying a simpler workflow. In the study, a comprehensive framework for whole-genome characterization using ONT was established and provided a useful tool for broad-scale analysis of FMDV-positive samples. However, these previous studies relied primarily on specific sample sets, including isolated viruses, clinical swabs, and environmental samples. Therefore, validating this protocol across a wider spectrum of field samples is crucial to ensure the robustness of the protocol under varying field conditions.

In March 2025, an FMD outbreak involving the O/ME-SA/Ind-2001e lineage was confirmed across 19 farms, including 14 cattle and 5 pig farms, in Jeollanam-do Province, Republic of Korea (27). From these affected sites, a total of 129 specimens were collected, representing a diverse range of sample types, including epithelium, vesicular fluid, serum, oral swabs, nasal swabs, and environmental samples. Notably, as the Republic of Korea maintains a nationwide vaccination policy for all cattle and pigs (28, 29), many of these specimens were characterized by reduced viral loads (30–34). In this study, we modified the ONT protocol described by Shaw et al. (26) to optimize sequencing performance. We then compared the sequencing depth between the original and modified protocols to evaluate the efficiency of our improvements. Furthermore, we performed a large-scale evaluation analyzing all clinical and environmental samples collected during the 2025 outbreak (*n* = 129) using the modified protocol. Finally, we examined the relationship between viral loads or sample types and sequencing metrics, identifying technical factors that influence ONT performance.

## MATERIALS AND METHODS

### Viruses

FMDV O/CJ/SKR/2023 and A/Gimpo/SKR/2018 were isolated in the Republic of Korea by the Animal and Plant Quarantine Agency (APQA). FMDV O/BAN11/2023 was isolated at the APQA from field samples provided by the Central Disease Investigation Laboratory in Bangladesh. The viral loads were standardized to cycle threshold (Ct) values of 24, 27, and 30 using a 10-fold serial dilution standard curve (*R*^2^ > 0.99; Table 1). All viruses were handled in a Biosafety Level 3 (BSL-3) containment facility at the APQA.

**TABLE 1:** Characteristics of FMDV strains used for validation

Strain	Accession no.	Cell line/passage	Target Ct	Titer (TCID_50_/mL)
O/CJ/SKR/2023	PP156917	BHK-21 (P3)	24/27/30	1.3 × 10^3^/1.3 × 10^2^/1.3 × 10^1^
O/BAN11/2023	PQ110577	BHK-21 (P5)	24/27/30	3.0 × 10^3^/3.0 × 10^2^/4.0 × 10^1^
A/Gimpo/SKR/2018	PZ575028	LFBK (P4)	24/27/30	5.0 × 10^3^/1.0 × 10^3^/2.0 × 10^2^

### Clinical and environmental sample collection

A total of 129 samples were collected from FMD-affected farms located in Jeollanam-do, Republic of Korea, between 14 March and 23 April 2025. These samples consisted of clinical specimens obtained from cattle and pigs (comprising epithelium samples, oral swabs, nasal swabs, serum samples, and a vesicular fluid sample) and environmental samples (collected by swabbing barns, vehicles, and feed within the affected sites; Table S1). The samples were transported to the laboratory in transport media (BD Diagnostics, NJ, USA). Epithelium samples were processed by homogenizing 50 mg of tissue in Dulbecco’s modified Eagle’s medium (Sigma-Aldrich, MO, USA) without fetal bovine serum (Gibco, NY, USA), using a bead-based homogenizer. All experimental procedures, including sample processing and lysis, were conducted in a BSL-3 facility at the APQA.

### RNA extraction and real-time RT-PCR

Viral RNA was extracted from all samples using the Nextractor NX-48S Viral NA kit (Genolution, Seoul, Republic of Korea) in accordance with the manufacturer’s protocol. The presence of FMDV RNA was detected using the PowerChek FMDV Multiplex Real-time PCR kit (3D/IRES; Kogenbiotech, Seoul, Republic of Korea) on a CFX96 System (Bio-Rad, CA, United States) in accordance with the manufacturer’s instructions. Ct values were recorded for all samples to assess viral load.

### cDNA synthesis

Reverse transcription was performed using the LunaScript Supermix (New England Biolabs, MA, USA) and the FMD_UNI_RT primer as described previously (26). Briefly, the reaction mixture consisted of 2 μL of LunaScript RT SuperMix (5×), 0.5 μL of the FMD_UNI_RT primer (50 pmol/μL), and 7.5 μL of the extracted RNA, in a total reaction volume of 10 μL. Thermal cycling was performed as follows: primer annealing at 25°C for 2 min, cDNA synthesis at 55°C for 10 min, and enzyme heat inactivation at 95°C for 1 min.

### PCR amplification

**Fig 1:**
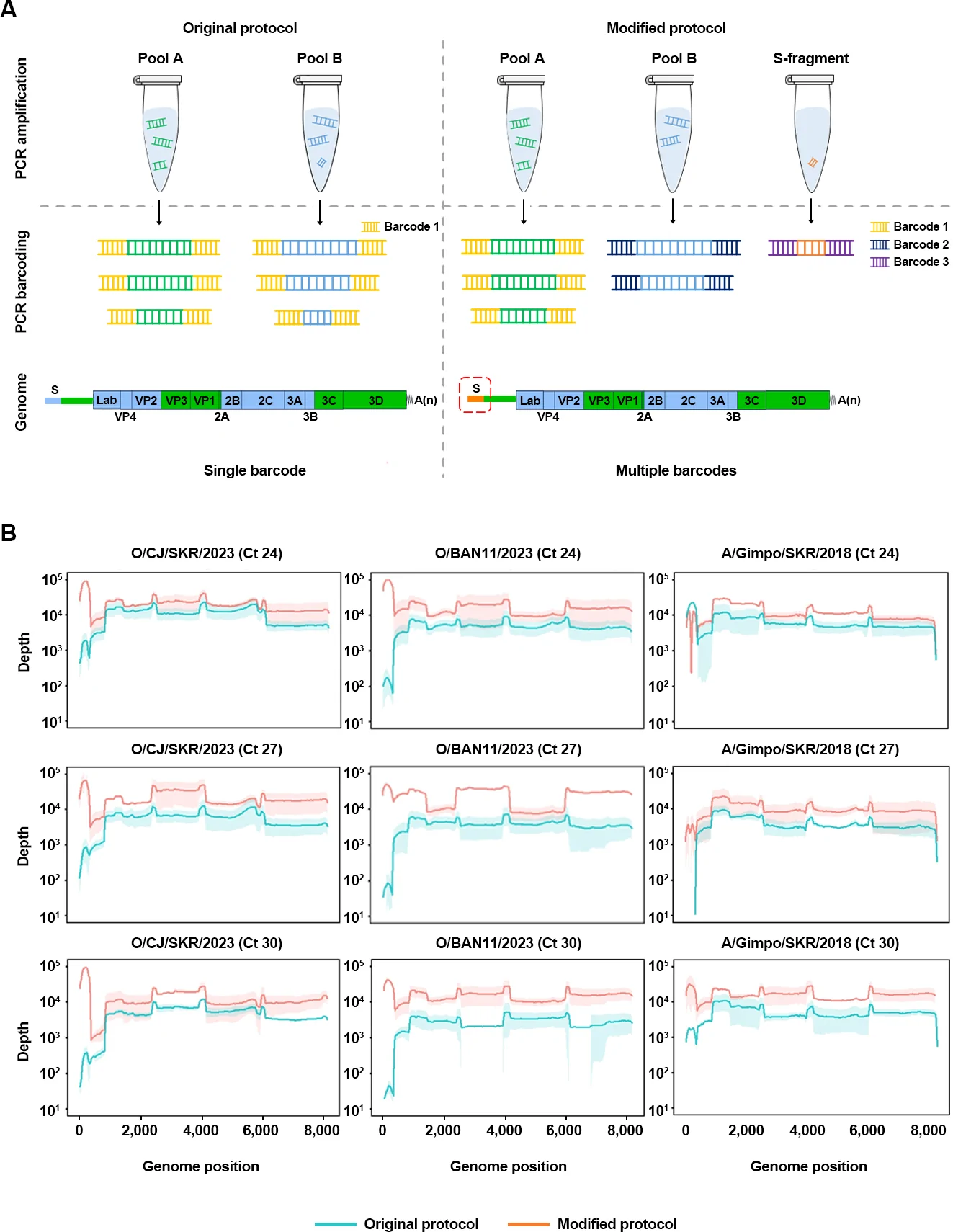
Independent amplification and barcoding of the S-fragment enhance sequencing depth across varying viral loads. (**A**) Schematic of the original and modified protocols. In the original protocol (left), genomes are amplified from Pools A and B and assigned a single barcode. In the modified protocol (right), the S-fragment is isolated from Pools A and B, and they are amplified in three distinct parts, with multiple barcodes assigned to each amplicon for nanopore sequencing. The red dashed box highlights the isolation of amplification and barcoding for the S-fragment. The genomic bar represents each pool, including the S-fragment (orange), Pool A (green), and Pool B (blue), in its respective region. (**B**) Comparison of whole-genome sequencing depth between the original and modified protocols using serial dilutions (Ct 24, 27, and 30) of three representative FMDV isolates: O/CJ/SKR/2023, O/BAN11/2023, and A/Gimpo/SKR/2018. Solid lines and shaded areas represent the mean depth and standard deviation, respectively, of independent triplicate experiments for the original (blue) and modified (orange) protocols. Whole-genome coverage plots display enhanced 5′-terminal S-fragment recovery under the modified protocol.

The entire FMDV genome was amplified using two separate strategies: the original scheme described by Shaw et al. (26) and a modified approach designed to enhance S-fragment amplification efficiency. Briefly, the original protocol involved dividing six overlapping fragments, covering the entire viral genome, into two pools, i.e., A and B, with each consisting of three amplicons. For the modified scheme, the genome was amplified in six genomic segments using primers divided into three pools: Pools A and B, and a separated S-fragment (using the specific S-fragment primers described by Shaw et al. (26) (Fig. 1A). Both amplification methods followed the reaction conditions described below. Reactions were performed using Platinum SuperFi II Green PCR Master Mix (Invitrogen, Carlsbad, CA, USA). The PCR reaction comprised 10 μL of PCR Master Mix, 8 μL of the primer pool (10 pmol/μL), and 2 μL of cDNA template, in a total volume of 20 μL. PCR was performed on a Mastercycler X50 thermal cycler (Eppendorf, Hamburg, Germany). The cycling conditions were adjusted, consisting of an initial denaturation at 98°C for 1 min, followed by 35 cycles of denaturation at 98°C for 10 s, annealing at 60°C for 15 s, and extension at 72°C for 3 min, with a final extension at 72°C for 3 min. The PCR products were quantified and sized using a 4150 TapeStation (Agilent Technologies, CA, USA). Samples were diluted to a final concentration of 20 ng/μL (200 ng total in 10 μL) for library preparation.

### Nanopore library preparation and sequencing

The DNA libraries were prepared using the Rapid Barcoding Kit 96 V14 (SQK-RBK114.96, Oxford Nanopore Technologies, Oxford, UK) in accordance with the manufacturer’s instructions. To evaluate library efficiency, two different barcoding strategies were compared. Briefly, in the original strategy (26), amplicons from Pools A and B were combined and tagged with a single barcode per sample (Fig. 1). In the modified strategy, Pools A and B and the S-fragment parts were separated, and three distinct barcodes were allocated to each sample. Each barcoding reaction involved incubating 10 μL of template DNA with 1.5 μL of rapid barcode at 30°C for 2 min, followed by 80°C for 2 min. The barcoded reactions were pooled and purified using AMPure XP beads (AXP; Beckman Coulter, CA, USA) at a 1:1 ratio. Subsequently, the pooled library was quantified and diluted to the recommended loading concentration prior to sequencing. The final library was loaded onto an R10.4.1 flow cell (FLO-MIN114) and sequenced on a MinION Mk1D device using the MinKNOW software (version 25.03.9). The sequencing run was conducted overnight (17–21 h). Field sample sequencing was performed across multiple MinION runs to accommodate multiplexing under the three-barcode strategy.

### Sequencing data processing and analysis

Basecalling was performed using Dorado (Oxford Nanopore Technologies, version 7.8.3) with a super-high accuracy algorithm, which is integrated into the MinKNOW software. A minimum quality score (*Q*-score) threshold of 10 was applied during the basecalling process. For data quality control, a filtering criterion was established to exclude low-quality reads. All reads with a mean quality score of less than 9 were classified into the “fastq_fail” folder and removed from the final data set used for further analyses. FASTQ files were imported into CLC Genomics Workbench 23.0.4 (QIAGEN, Carlsbad, CA, USA) for downstream analysis. After demultiplexing, reads from the three barcodes assigned to each sample (Pool A, Pool B, and the S-fragment) were merged into a single sequence list per sample prior to reference mapping. Reads were mapped to the complete genome sequence of an early isolate from the 2025 outbreak using the “Map Long Reads to Reference” tool. Consensus sequences were extracted and aligned with a reference sequence using the ClustalW algorithm in BioEdit 7.7.1 (35). In this study, a complete genome was defined as retaining 100% coverage of the whole genome region at a sequencing depth of > 50×. Similarly, a complete VP1 was defined as attaining 100% coverage of the VP1 coding region with the same depth threshold. Consensus sequences were generated for coverage assessment purposes rather than for variant-level analysis. For the protocol comparison experiments, polished consensus sequences were generated using the NanoFMDV pipeline (26), which is publicly available on GitHub (https://github.com/rjorton/nanofmdv). Coverage depth was calculated via samtools (36) and visualized as the mean sequencing depth with standard deviation at each nucleotide position using the ggplot2 package in R (version 4.5.2) (37).

### Illumina sequencing and data processing

The three validation strains and selected representative field samples were analyzed using an Illumina sequencing platform to validate the nanopore-derived consensus sequences. Briefly, libraries were prepared using QIAseq Single Cell RNA Library Kit (Cat. No. 180705; QIAGEN, Hilden, Germany) in accordance with the manufacturer’s instructions (38) and sequenced on an iSeq 100 (Illumina, San Diego, CA, USA), generating 2 × 150 bp paired-end reads. The raw Illumina reads were quality filtered and trimmed using CLC Genomics Workbench 23.0.4 (QIAGEN), and the clean reads were mapped to the complete genome sequence of an early FMDV isolate from the 2025 outbreak as the reference sequence. Consensus sequences were generated for comparison with the nanopore-derived consensus sequences. Pairwise nucleotide identity between the Illumina- and nanopore-derived consensus sequences was calculated using CLC Genomics Workbench 23.0.4 to assess concordance.

### Statistical analysis

Differences in sequencing success rates among sample types and Ct groups were evaluated using Pearson’s *χ*^2^ test. Sequencing depths across Ct groups were compared using one-way analysis of variance (ANOVA), followed by Tukey’s post-hoc test. The relationship between viral loads and sequencing metrics was quantified using Spearman rank correlation analysis. All statistical analyses were performed using GraphPad Prism software (version 8.0.1; GraphPad Software, San Diego, CA, USA). In all analyses, *P*-values less than 0.05 were considered to indicate statistical significance.

## RESULTS

### Sequencing depth comparison for the S-fragment using original and modified protocols

To compare the efficiency of the original and modified protocols, we designed a revised barcoding strategy involving independent S-fragment processing (Fig. 1A). In this method, we amplified the entire genome into six segments using three distinct pools: we assigned Pools A and B, and the S-fragment amplicons distinct barcodes, whereas the original protocol combined Pools A and B under a single barcode per sample. To validate this methodology, we analyzed the serial dilutions of three representative isolates (O/CJ/SKR/2023, O/BAN11/2023, and A/Gimpo/SKR/2018) at Ct values 24, 27, and 30. The modified protocol markedly improved S-fragment recovery across nearly all tested strains (Fig. 1B). For both the O/CJ/SKR/2023 and O/BAN11/2023 strains, sequencing depth remained consistently higher than the original protocol across all dilution levels. While the original protocol exhibited higher depth than the modified for the A/Gimpo/SKR/2018 strain at Ct 24, this efficiency declined sharply at lower viral loads. By contrast, the modified protocol maintained a stable and robust sequencing depth even at Ct 27 and 30, indicating that S-fragment isolation during the amplification and barcoding steps effectively mitigates pooling-related biases, thereby ensuring consistent genomic recovery even in samples with reduced viral loads. Furthermore, the consensus sequences obtained using the modified protocol were completely identical to those generated by Illumina sequencing (Table S2). Based on these results, we adopted this modified protocol to analyze all clinical and environmental samples from the 2025 outbreak.

### Sample type-dependent differences in success rates

**Fig 2:**
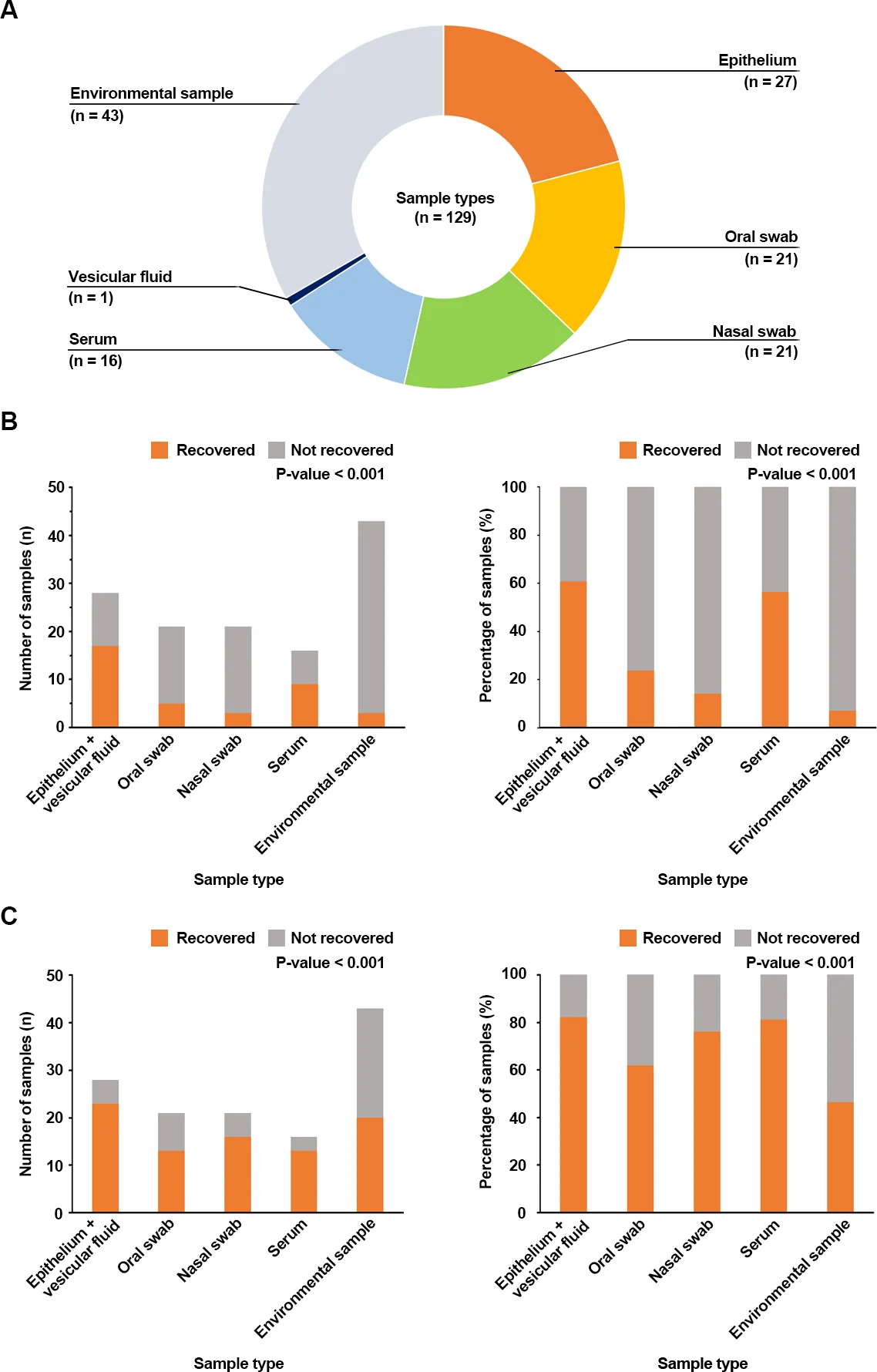
Nanopore sequencing success depends on sample type. (**A**) FMDV-positive sample distributions by sample type. Whole-genome (**B**) or VP1 coding region (**C**) recovery analyses: left panel, number of “Recovered” (orange) and “Not recovered” (gray) samples; right panel, proportional success rate for each sample type. *P*-values denote significant differences between sample types, as determined by Pearson’s *χ*^2^ test (*P* < 0.001).

Nineteen farms in the Republic of Korea were confirmed FMD positive in 2025. We collected a total of 129 various samples that tested positive using 3D real-time RT-PCR assay from officially reported FMD-infected farms, comprising six different types: 27 epithelium samples, 21 oral swabs, 21 nasal swabs, 16 serum samples, 1 vesicular fluid sample, and 43 environmental samples (Fig. 2A). Whole-genome sequencing success rates (recovery rate) varied significantly depending on sample type (Pearson’s *χ*^2^ test, *P* < 0.001; Fig. 2B). The epithelium and vesicular fluid samples (60.7%, *n* = 17) and serum (56.3%, *n* = 9) samples showed the highest recovery rates, whereas the oral swabs (23.8%, *n* = 5), nasal swabs (14.3%, *n* = 3), and environmental samples (7.0%, *n* = 3) exhibited lower sequence recovery. We observed a similar trend for the VP1 coding region, although the proportion of recovered samples for the VP1 region was higher compared with that for WGS (Pearson’s *χ*^2^ test, *P* < 0.001; Fig. 2C). The epithelium and vesicular fluid samples (82.1%, *n* = 23) and serum samples (81.3%, *n* = 13) again resulted in the highest rates of sequence recovery. The success rates of the oral and nasal swabs (61.9% and 76.2%, respectively) were lower than those of the epithelium and vesicular fluid samples and serum samples. Environmental samples showed the lowest success rate at 46.5% (*n* = 20). Additionally, the WGSs obtained from selected clinical samples (*n* = 4) showed perfect consistency with the sequences generated using the Illumina platform (Table S2).

### Sample type- and viral load-dependent sequence qualities

**Fig 3:**
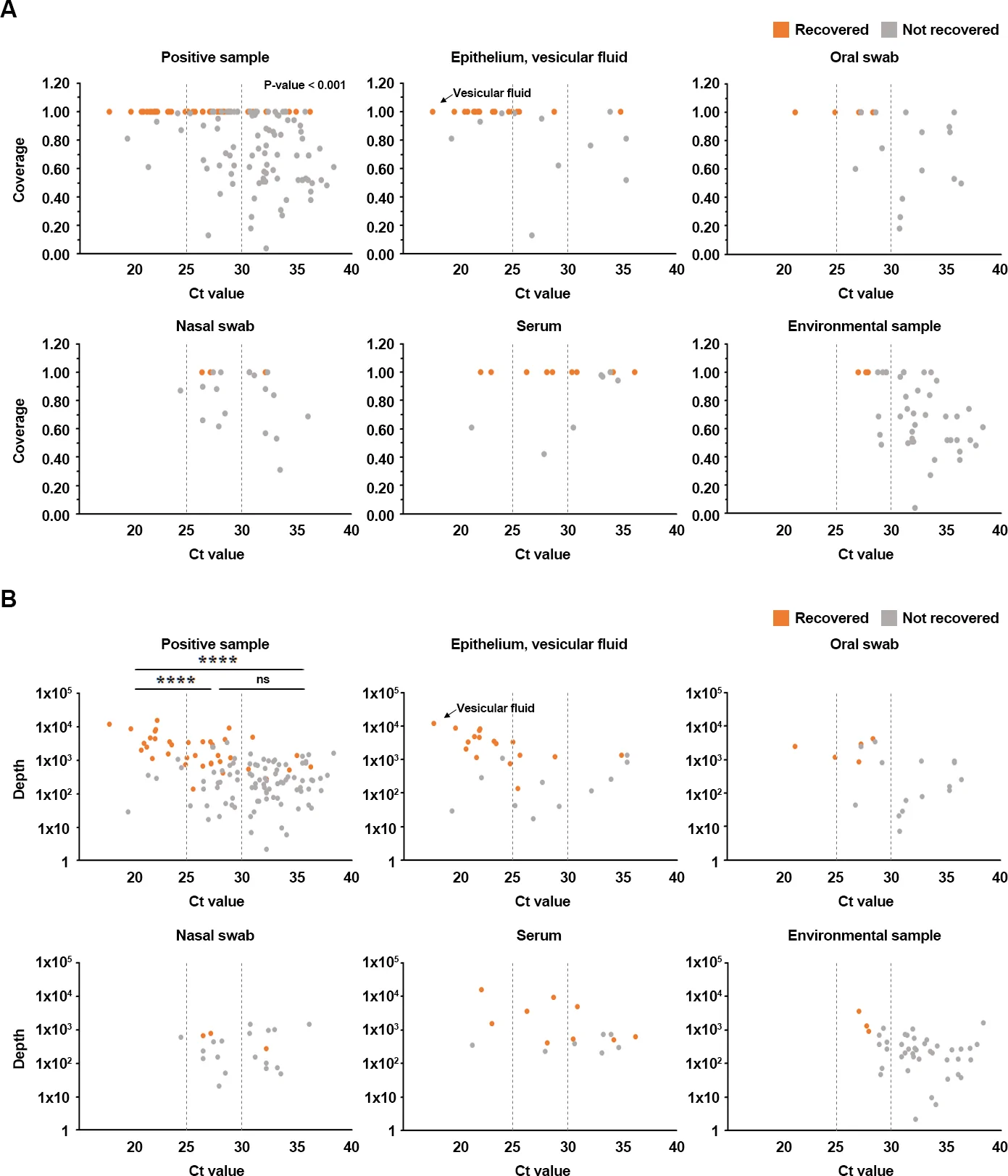
Sequencing quality exhibits an inverse trend relative to viral loads across diverse sample types. (**A**) Relationship between Ct values and WGS coverage. The scatter plots present genome coverages relative to Ct values for all 129 positive samples tested, stratified by sample type. Coverage and Ct range were compared by a Pearson’s *χ*^2^ test (*P* < 0.001). (**B**) Relationship between Ct values and sequencing depth. The plots display the sequencing depth achieved for samples with varying Ct values. Differences in sequencing depth among Ct ranges were evaluated by one-way ANOVA (****P < 0.0001, ns, not significant). In all panels, the data points are colored by WGS success status (orange: complete genome, defined as 100% coverage at > 50× depth; gray: partial-genome recovery).

To further analyze factors affecting sequencing success, we investigated the association between viral load and sequencing quality metrics (i.e., genome coverage and sequencing depth) in the various sample types. Ct distribution analysis revealed that the epithelium and vesicular fluid samples contained the highest viral loads, with 57.1% with a Ct ≤ 25. By contrast, the other sample types mostly exhibited reduced viral loads (Ct > 30), especially the environmental samples (76.7%). Overall, 53.5% of all samples had Ct values above 30, whereas only 17.1% were at Ct ≤ 25 (Table S3). Viral load distributions varied considerably across different sample types, directly influencing the overall success rates (Pearson’s *χ*^2^ test, *P* < 0.001; Fig. 3A). We observed an inverse trend between Ct values and genome coverage, defined as achieving 100% WGS coverage. Across most sample types, we recovered sequences in the samples with the highest viral load (Ct ≤ 25), resulting in a 77.3% total recovery rate (66.7%–100%; Table S4). The influence of sample matrix became increasingly pronounced at lower viral titers. In the intermediate range (25 < Ct ≤ 30), the serum (75%), epithelium, and vesicular fluid (42.9%) samples outperformed the swab-based samples, which showed a notable drop in recovery efficiency (22.2%–37.5%). The most pronounced differences were observed in the low-titer category (Ct > 30). Although the recovery rates of the oral swabs and environmental samples fell to 0%, those of the serum samples remained robust, maintaining a 44.4% WGS recovery rate, which was significantly higher than that of the epithelium, vesicular fluid, and nasal swabs (20% and 9.1%, respectively). Figure 3B presents the sequencing depth analysis relative to the Ct values. Consistent with the coverage results, sequencing depth significantly varied across Ct ranges (one-way ANOVA, *P* < 0.0001), showing an inverse trend between Ct values and sequencing depth. The mean sequencing depth decreased from 4,026× in the high-viral-load group (Ct ≤ 25) to 1,466× in the intermediate range (25 < Ct ≤ 30) and further to 498× for the low-titer samples (Ct > 30). Depth also varied across sample types; in specific, the serum, epithelium, and vesicular fluid samples maintained higher sequencing depths (mean depths of 4,205× and 4,070×, respectively) than the swab-based samples, e.g., oral swabs, environmental samples, and nasal swabs (mean depths of 2,393×, 2,035×, and 600×, respectively). This pattern was especially evident in the low-titer samples (Ct > 30), with epithelium and serum showing mean depths of 1,424× and 1,709×, respectively, compared with 283× for nasal swabs.

### Inconsistent amplification efficiency in the samples that failed whole-genome sequencing

**Fig 4:**
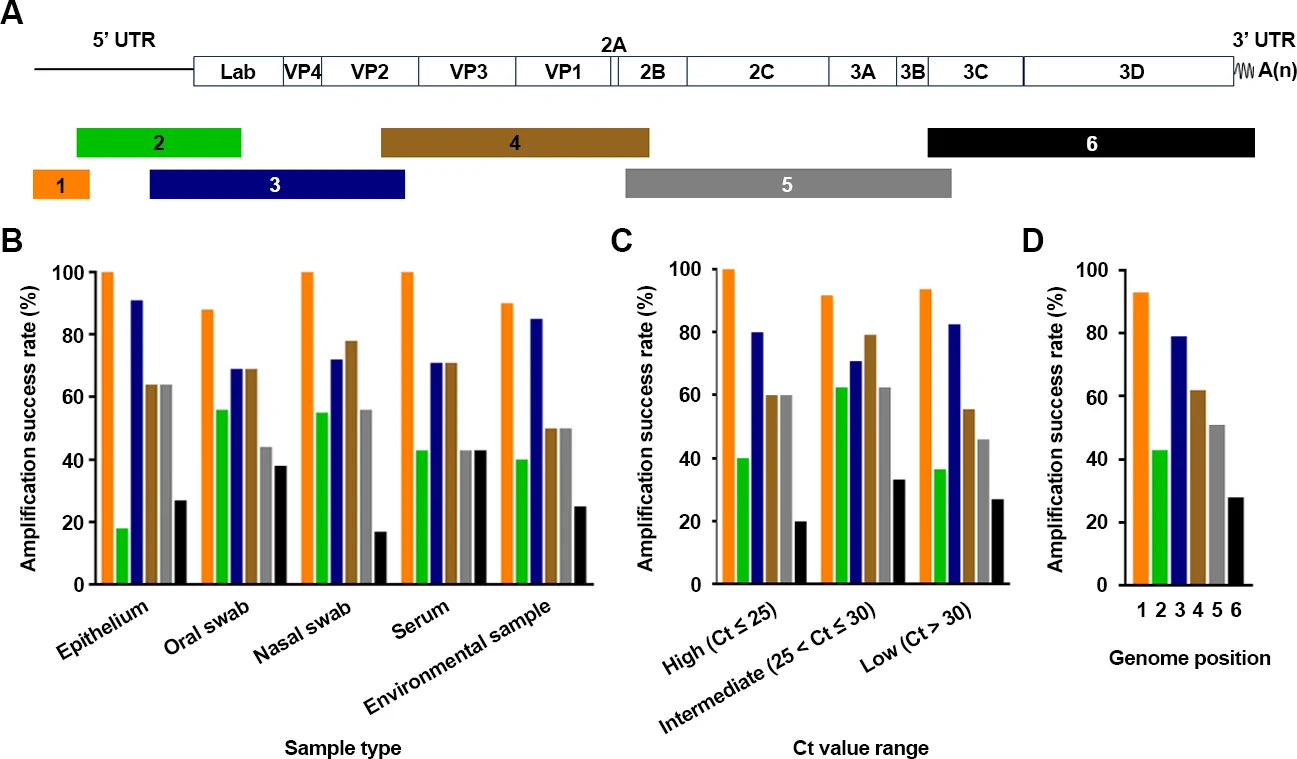
Amplicon amplification efficiency varies depending on genomic position but is consistent across the sample types. (**A**) Schematic of the FMDV genome and six overlapping amplicons. Amplicons are numbered 1–6 (5′–3′) according to their genomic positions. (**B and C**) Amplification success rates for amplicons 1–6 categorized by sample types (**B**) and Ct value ranges (**C**). (**D**) Overall amplification success rates based on genome position, summarizing the performance of each amplicon (1–6) across the 92 samples that failed to achieve complete WGS recovery.

To identify the specific WGS recovery-limiting genomic regions, we analyzed the amplification success rates for each of the six overlapping amplicons, specifically among the 92 samples that failed whole-genome sequencing (data not shown). These six segments, designated as amplicons 1–6, represented the overlapping genomic regions covering from the 5′-untranslated region (5′-UTR) to the 3′-UTR (Fig. 4A). When analyzed by sample type, the epithelium and serum samples as well as nasal swabs exhibited a 100% success rate for amplicon 1, validating the efficacy of the separate amplification strategy for the S-fragment (Fig. 4B). By contrast, we observed a clear positional bias in the remaining regions. For the epithelium samples, nasal swabs, and environmental samples, amplicons 2 and 6 yielded the lowest recovery. Specifically, in the epithelium samples, although the recovery rate for amplicon 2 (18%) was slightly lower than that for amplicon 6 (27%), both performed worse than the other four amplicons. We observed a different trend in oral swabs, where amplicons 6 (38%) and 5 (44%) displayed the lowest success rates. For the serum samples, amplicons 2, 5, and 6 exhibited equally poor success rates. To determine amplification efficiency depending on viral loads, the success rates of the amplicons were evaluated (Fig. 4C). Amplicons 2 and 6 consistently yielded the lowest recovery in both high- and low-viral-load groups, whereas amplicons 2, 5, and 6 remained the most challenging in the intermediate range. Aggregating these results across all samples confirmed a significant positional bias relative to the genome position (Fig. 4D). Amplicon 1 achieved the highest recovery rate of 93.5%, consistent with S-fragment optimization in the modified protocol (Fig. 1B and 4D). By contrast, the recovery rates reached their lowest level in amplicon 6 (28%), followed by amplicon 2 (43%). Consequently, we identified uneven amplification efficiency, specifically poor recovery of amplicons 2 and 6, as the primary factor limiting whole-genome sequencing success in the clinical and environmental samples.

## DISCUSSION

The 2025 FMD outbreak in the Republic of Korea posed a distinct diagnostic challenge compared with the previous outbreak in 2023. While positive cases in 2023 were identified almost exclusively from the epithelial samples and nasal swabs, the 2025 outbreak involved a wider sample type diversity, including vesicular fluid, oral swabs, and environmental samples that were identified as FMDV positive using real-time RT-PCR. The fact that the vaccination policy has been implemented nationwide in the Republic of Korea since 2010 further compounded this complexity (28, 29, 39). Consistent with both experimental and field observations, vaccination significantly reduces viral shedding and transmission across various livestock species (30–33). The present study clearly highlighted these suppressive effects (Table S3). Specifically, more than half of all FMDV-positive samples (53.5%) had real-time RT-PCR Ct values > 30, indicating that low-viral-load specimens are common in settings where vaccination is implemented. Consequently, improving the analytical sensitivity of diagnostic methods and genomic analysis was a key motivation for this work.

To address this aspect, we applied an optimized amplicon-based nanopore sequencing workflow. We selected this PCR-based approach, similar to the L_scheme protocol previously designed (26), for its analytical sensitivity and broad FMDV serotype coverage. Although PCR-free direct sequencing approaches offer unbiased results, they retain lower analytical sensitivity to detect viral genomes in samples with reduced viral load and without extensive enrichment (40, 41). We assessed the suitability of the modified protocol to sequence FMD viruses from a diverse set of field samples (*n* = 129) across a wide range of Ct values, providing insights into the most optimal sample types for molecular analysis during an FMD outbreak. Using this approach, we successfully recovered WGSs (28.7%) and sequences for VP1 coding regions (65.9%) from the clinical and environmental samples.

An optimized library preparation step was one of the key technical improvements in our study. During early testing, the S-fragment sequences were often absent or exhibited very low depth, possibly because they were outcompeted by longer amplicons during the pooling process. This issue was resolved by processing the S-fragment independently for both PCR amplification and barcoding. Although individual PCR for each amplicon can theoretically reduce primer interference, it would substantially increase the number of PCR reactions, reagent use, hands-on workload, and library preparation complexity when applied to large field-sample sets. Therefore, we separated only the consistently underrepresented S-fragment for independent amplification and barcoding while retaining multiplex amplification for the remaining genomic regions. This three-pool design improved the recovery of the 5′ genomic region while preserving the practical advantages of the original workflow. While previously published approaches struggled with low sequencing depth (< 10×) in this region (26), our modified protocol achieved a recovery rate exceeding 93.5% for amplicon 1. As the S-fragment forms part of the 5′-UTR, which is critical for viral replication (42), ensuring its reliable recovery is a major advantage.

In this study, we verified the single-base accuracy of our method by parallel sequencing using the Illumina platform for seven representative samples. It could support the reliability of our findings for downstream molecular analysis, and it is consistent with previous reports on the high consensus fidelity of nanopore sequencing for viral genomes (24, 43). Although parallel Illumina sequencing could not be performed for serum and environmental samples due to insufficient sample volumes during the active outbreak response, the high sequencing depth (at least 144×) achieved across these matrices supports the high accuracy of our nanopore-derived sequences.

Independent triplicate experiments were performed from initial RNA extraction to sequencing for viral loads (Ct 24, 27, and 30) presented in Fig. 1B to ensure technical reproducibility and workflow robustness. Additionally, rigorous quality control was maintained through the strict inclusion of negative controls, which exhibited no detectable amplification during real-time RT-PCR and an absence of bands via capillary electrophoresis. These combined measures effectively rule out cross-contamination and pre-analytical errors, confirming the stability and reliability of the optimized protocol for reduced viral load genomic analysis.

Our comparative analysis of sample types offers practical guidance for field strategies. Our results revealed a significant association between sequencing success rates and sample type (Fig. 2). Vesicular fluid and epithelium samples yielded the highest quality data, confirming their suitability as primary specimens for FMD diagnostics and sequencing. The robustness of the method to test sera was particularly noteworthy. Although sequencing success in the swab samples declined sharply at real-time RT-PCR Ct values above 30, the serum samples maintained higher sequencing coverage and depth than the other sample types (Fig. 3). This divergence in sequencing success, even at equivalent Ct values, is possibly caused by the different compositions of these matrices (44) and may reflect factors such as the presence of host nucleic acids, PCR inhibitors, and scope for nucleic acid degradation. In addition, swab and environmental samples may contain abundant background material, which can hinder effective target recovery in low-titer specimens. Swab-based samples (e.g., oral swabs, nasal swabs, and environmental samples) are characterized by an overwhelming host DNA abundance, creating significant competition during library preparation and reducing viral detection efficiency (44, 45). By contrast, we speculate that serum exhibits a relatively lower host-to-virus nucleic acid ratio, allowing for more efficient target enrichment and superior genomic coverage. Notably, in this study, we successfully recovered the WGS from serum samples with Ct values > 30, whereas previous studies did not report full genome coverage for samples in this Ct range (26). Although a sequencing depth of 30× to 50× is generally considered the minimum threshold for consensus sequence generation (8, 46), higher depths provide superior genomic resolution (47). These findings support prioritizing serum as a sample type for cases with reduced viral loads. In addition, although this study included both cattle- and pig-derived specimens, all WGSs were exclusively recovered from cattle. This result was attributed to the relatively high mean Ct value (33.68) observed in the pig-derived samples, which limited consistent target enrichment for whole-genome sequencing. A detailed analysis of VP1 sequence recovery rates and sequencing depth for these pig-derived samples will be conducted in future research. Furthermore, VP1 sequence recovery from environmental samples (47%) highlights their potential as a non-invasive monitoring option. Environmental samples, such as swabbing of barn surfaces and feed, are typically challenging to characterize because of low viral abundance and poor sample quality (48, 49). However, our results demonstrate that nanopore sequencing can effectively retrieve genomic information even from these difficult sources (Fig. 2 and 3). Notably, our analysis revealed high sequence identity (99.5%–100%) for VP1 between these environmental sequences and the isolates from the corresponding farms. Moreover, the high nucleotide identity (99.6%–100%) of the WGSs of them would provide stronger evidence to rule out cross-contamination, although most environmental samples yielded VP1 sequences. These findings support the potential use of environmental samples as complementary genetic evidence during outbreak investigations, while highlighting that their performance remains constrained by viral load and sample quality (50–52).

WGS success rates were significantly influenced by Ct ranges, and sequencing depth significantly varied across Ct ranges (Fig. 3A and B). Although we attempted to perform a statistical comparison of sequencing depth within Ct ranges for each sample type, the limited sample size in sub-categories precluded a definitive sub-group analysis. Although sequencing depth was also examined by sample type within each Ct category, several subgroups contained insufficient samples for reliable statistical comparison. No significant difference in sequencing depth was detected between the intermediate- (25 < Ct ≤ 30) and low-titer (Ct > 30) groups, but this result should be interpreted cautiously because limited sample sizes and matrix-related variability may have reduced statistical power. Spearman’s rank correlation analysis showed moderate negative correlations between Ct values and sequencing metrics (Spearman’s *P* = −0.41 to −0.50), supporting the expected decline in genome coverage and sequencing depth at higher Ct values. These results indicate that viral load is a major determinant of sequencing success, while sample matrix and amplicon-specific amplification efficiency may also affect sequencing outcomes.

Although the modified protocol performed generally well, we encountered specific obstacles that prevented recovery of complete WGS in certain cases. Except for amplicon 1, which consistently achieved high recovery across all sample types, the amplification efficiency for the other PCR targets varied depending on the sample type, possibly because of the quality of target nucleic acids and the effect of PCR inhibitors inherent to each sample matrix (44). Notably, the recovery rates of amplicons 2 and 6 were consistently low regardless of both viral load and sample type, with inefficient efficiency persisting even in high-titer samples (Ct ≤ 25; Fig. 3). Since our workflow relies on an amplicon-based approach, the fact that this failure was independent of sample conditions indicates that the failure is primarily driven by primer-dependent limitations such as unsuitable binding sites or design constraints within these specific genomic regions rather than a simple detection limit issue. The failure of amplicon 2 is possibly due to the poly(C) tract at the 5′ end, reportedly interfering with primer binding and polymerase activity (53, 54). Furthermore, the region immediately downstream of the poly(C) region is highly diverse, complicating the design of the primer set. The failure of amplicon 6 is possibly due to its length (2.2 kb), representing the longest amplicon in our scheme and resulting in lower PCR amplification efficiency (55). This enrichment strategy is less suited for resolving complex viral recombination or quasispecies, representing an important objective for further study; nevertheless, it ensures robust consensus accuracy for epidemiological tracking. Future studies could focus on exploring alternative primer designs to overcome the amplification reduction associated with the 5′-UTR structure for amplicon 2 and splitting amplicon 6 into smaller, overlapping segments. These additional optimization steps may substantially enhance the recovery of WGS, enabling comprehensive genomic analysis even from challenging field samples.

Although the primer set used in this workflow was originally designed for broad-spectrum FMDV amplification (26), the present field evaluation was based on samples collected during a single outbreak involving the O/ME-SA/Ind-2001e lineage in the Republic of Korea. In addition, the protocol comparison experiments included representative serotype O and A viruses, but the remaining FMDV serotypes and broader lineage diversity were not directly evaluated in this study. Therefore, the findings should be interpreted as evidence supporting the potential utility of the optimized workflow under the outbreak conditions tested here, rather than as proof of universal applicability across all FMDV serotypes, lineages, or field settings. Additional validation using more diverse serotypes, lineages, geographic regions, and outbreak contexts will be required in further study.

This study represents the first large-scale validation of an optimized nanopore sequencing workflow during an active FMD outbreak in the Republic of Korea. Our findings demonstrate that this platform mitigates key diagnostic challenges associated with reduced viral loads in vaccinated populations. By isolating the S-fragment during library preparation, we achieved robust genomic recovery and sequencing depth for reliable genome assembly, particularly in serum samples, which proved superior to swab-based methods. These results confirm that nanopore sequencing is a valuable tool for generating timely genomic data to support emergency response. Although we performed this study using samples collected during a single outbreak in the Republic of Korea, where nationwide vaccination was implemented, our results highlight trends that may provide genomic diagnostic information in similar vaccinated settings. Further evaluation using samples from different geographic regions and surveillance systems would be required to assess broader applicability.

## Data Availability

Representative whole-genome and VP1 sequences generated in this study have been deposited in the NCBI GenBank repository under accession numbers PZ575027 (WGS) and PZ575028 (VP1). All associated metadata and study details required to replicate the findings are provided within the manuscript and its supplemental material.

## SUPPLEMENTAL MATERIAL

Table S1 spectrum.00791-26-s0001.xlsx Metadata of the 129 FMDV-positive field samples.

Table S2 spectrum.00791-26-s0002.docx Comparison of whole-genome sequences analyzed using Illumina and Nanopore platform.

Table S3 spectrum.00791-26-s0003.docx Distribution of viral load proportions in each sample type.

Table S4 spectrum.00791-26-s0004.docx Sample type- and Ct value-dependent whole genome and VP1 region sequencing success rates.

ASM does not own the copyrights to Supplemental Material that may be linked to, or accessed through, an article. The authors have granted ASM a non-exclusive, world-wide license to publish the Supplemental Material files. Please contact the corresponding author directly for reuse.
