# Supplementary material for: Evaluation of amplicon-based nanopore sequencing for foot-and-mouth disease viruses in clinical and environmental samples
Source: Microbiol Spectr. 2026 Jul 28;14(9):e00791-26. doi: 10.1128/spectrum.00791-26 (PMC13532212; doi:10.1128/spectrum.00791-26)
Supplement: Supplementary file 1 [file spectrum.00791-26-s0002.docx]

**Table S2.** Comparison of whole-genome sequences analyzed using Illumina and Nanopore platform

| **Sample types (Strain/Sample ID)** | **Mean depth (**×) | | **Concordance (%)^*^** | **SNP difference^*^** | **IN/DEL difference^*^** |
| --- | --- | --- | --- | --- | --- |
|  | **Illumina** | **Nanopore** |  |  |  |
| Virus isolate (O/CJ/SKR/2023) | 7,305 | 20,833 | 100 | 0 | 0 |
| Virus isolate (O/BAN11/2023) | 1,326 | 30,469 | 100 | 0 | 0 |
| Virus isolate (A/Gimpo/SKR/2018) | 2,676 | 14,921 | 100 | 0 | 0 |
| Vesicular fluid (#12-2) | 3,731 | 12,572 | 100 | 0 | 0 |
| Epithelium (#3-1-13) | 2,216 | 9,317 | 100 | 0 | 0 |
| Epithelium (#8-2) | 4,216 | 2,179 | 100 | 0 | 0 |
| Epithelium (#9-3) | 156 | 3,455 | 100 | 0 | 0 |

^*^analyzed excluding poly(A) and poly(C)
