# Supplementary material for: Evaluation of amplicon-based nanopore sequencing for foot-and-mouth disease viruses in clinical and environmental samples
Source: Microbiol Spectr. 2026 Jul 28;14(9):e00791-26. doi: 10.1128/spectrum.00791-26 (PMC13532212; doi:10.1128/spectrum.00791-26)
Supplement: Supplementary file 2 [file spectrum.00791-26-s0003.docx]

**Table S3.** Distribution of viral load proportions in each sample type

|  | **Range of Ct values** | | |
| --- | --- | --- | --- |
| **Sample types** | **Ct^*^ ≤ 25** | **25 < Ct ≤ 30** | **Ct > 30** |
| Epithelium + vesicular fluid (n = 28) | 57.1% (n = 16) | 25.0% (n = 7) | 17.9% (n = 5) |
| Oral swab (n = 21) | 9.5% (n = 2) | 38.1% (n = 8) | 52.4% (n = 11) |
| Nasal swab (n = 21) | 4.8% (n = 1) | 42.9% (n = 9) | 52.4% (n = 11) |
| Serum (n = 16) | 18.8% (n = 3) | 25.0% (n = 4) | 56.3% (n = 9) |
| Environmental sample (n = 43) | - | 23.3% (n = 10) | 76.7% (n = 33) |
| Total samples (n = 129) | 17.1% (n = 22) | 29.5% (n = 38) | 53.5% (n = 69) |

^*^ Ct value in real-time RT-PCR targeting 3D
