# Supplementary material for: Evaluation of amplicon-based nanopore sequencing for foot-and-mouth disease viruses in clinical and environmental samples
Source: Microbiol Spectr. 2026 Jul 28;14(9):e00791-26. doi: 10.1128/spectrum.00791-26 (PMC13532212; doi:10.1128/spectrum.00791-26)
Supplement: Supplementary file 3 [file spectrum.00791-26-s0004.docx]

**Table S4.** Sample type- and Ct value- dependent whole genome and VP1 region sequencing success rates (%)

| **Sample types** | **Whole genome** | | |  | **VP1 region** | | |
| --- | --- | --- | --- | --- | --- | --- | --- |
|  | **Ct^*^ ≤ 25** | **25 < Ct ≤ 30** | **Ct > 30** |  | **Ct ≤ 25** | **25 < Ct ≤ 30** | **Ct > 30** |
| Epithelium + vesicular fluid (n = 28) | 81.3% (13/16) | 42.9% (3/7) | 20.0% (1/5) |  | 93.8% (15/16) | 71.4% (5/7) | 60.0% (3/5) |
| Oral swab (n = 21) | 100.0% (2/2) | 37.5% (3/8) | 0.0% (0/11) |  | 100.0% (2/2) | 75% (6/8) | 45.5% (5/11) |
| Nasal swab (n = 21) | 0.0% (0/1) | 22.2% (2/9) | 9.1% (1/11) |  | 0.0% (0/1) | 88.9% (8/9) | 72.7% (8/11) |
| Serum (n = 16) | 66.7% (2/3) | 75.0% (3/4) | 44.4% (4/9) |  | 66.7% (2/3) | 75.0% (3/4) | 88.9% (8/9) |
| Environmental sample (n = 43) | - | 30.0% (3/10) | 0.0% (0/33) |  | - | 80% (8/10) | 36.4% (12/33) |
| Total samples (n = 129) | 77.3% (17/22) | 36.8% (14/38) | 8.7% (6/69) |  | 86.4% (19/22) | 79.0% (30/38) | 52.2% (36/69) |

^*^ Ct value in real-time RT-PCR targeting 3D
